# Vaccination Coverage and Attitudes in Children and Adults on Biologic Therapies: Cocooning Strategies, Undervaccination Factors and Predictors of Favorable Attitudes

**DOI:** 10.3390/vaccines13020152

**Published:** 2025-02-01

**Authors:** Charikleia Kariniotaki, George Bertsias, Emmanouil Galanakis, Chrysoula Perdikogianni

**Affiliations:** 1 Department of Pediatrics, University Hospital of Heraklion, Medical School, University of Crete, 71003 Heraklion, Greece; med9p1110059@med.uoc.gr (C.K.); emmgalan@uoc.gr (E.G.); 2 Department of Rheumatology and Clinical Immunology, University Hospital of Heraklion, Medical School, University of Crete, 71003 Heraklion, Greece; gbertsias@uoc.gr

**Keywords:** vaccination status, biologic agents, cocooning strategy, infectious diseases

## Abstract

Background: Infections pose a significant risk of morbidity and mortality to patients on biologics, with the vaccination of both patients and their close contacts serving as a key preventive measure. Despite its importance, there are limited data on the vaccination coverage for this group, and no studies have examined the vaccination status of patients’ close contacts. Objectives: To assess vaccination rates among patients on biologics and their household contacts, identifying reasons for inadequate vaccination and examining factors influencing vaccination status and attitudes is crucial. Methods: A cross-sectional study was conducted from September 2022 to February 2023 at the two hospitals in Heraklion, Crete, including adult and pediatric patients on biologics. Data were collected through medical records and interviews and analyzed using Microsoft Excel 2016 and MedCalc2006. Results: Among the 446 adults, vaccination rates were as follows: 83% for COVID-19, 73.8% for influenza, 64.5% for the pneumococcal conjugate vaccine, 29.6% for the pneumococcal polysaccharide vaccine, and 4% for Tdap. Among the 26 children included, those with basic immunization schedule coverage exceeded 96%, but rates for the vaccines usually administered at adolescence were lower (Tdap: 47.8%, HPV: 42.1%, MenACWY: 66.7%). COVID-19 vaccination was at 38.5%. Regarding the additional vaccines recommended due to treatment-induced immunosuppression, 69.2% of pediatric patients received the annual influenza vaccine, while only 19.2% received the pneumococcal polysaccharide vaccine. Household contacts demonstrated low vaccination rates (<59%), except for COVID-19 (81%). Female gender (*p* < 0.007) and older age (by 1 year, *p* < 0.001) were associated with favorable attitudes and higher coverage in adults, while in pediatric patients, no statistically significant associations were found. A lack of physician recommendation was the primary reported reason for not being vaccinated. Conclusions: Significant vaccination gaps exist among patients on biologics and their close contacts, largely due to inadequate physician recommendations. Raising awareness and strengthening healthcare provider roles are essential to improve coverage in this high-risk group.

## 1. Introduction

Biologic agents are increasingly used as immunomodulators, often in combination with other immunosuppressive drugs, for the management of various chronic inflammatory conditions. These include juvenile idiopathic arthritis, rheumatoid arthritis, ankylosing spondylitis, lupus, inflammatory bowel disease, and other autoimmune and autoinflammatory disorders. Their use has expanded significantly in recent years, reflecting advancements in treatment strategies and growing recognition of their efficacy. These agents primarily take the form of monoclonal antibodies targeting pro-inflammatory cytokines, along with small molecules like Janus kinase (JAK) inhibitors [1]. Biologic therapies have been shown in randomized control trials and clinical practice to effectively delay disease progression, alleviate pain and edema, and improve quality of life in patients with arthritis, skin conditions, and inflammatory bowel disease [2].

However, their immunosuppressive effect increases the risk of infection, necessitating additional vaccinations for these patients [3]. Also, due to their immunomodulatory effect that persists throughout treatment and even for weeks post-treatment, it is advised that patients complete their vaccination schedules before initiating therapy. While on treatment, patients can safely receive all inactivated vaccines, but live vaccines are generally contraindicated for a period of at least 3–6 months following the cessation of treatment due to the possible risk of infection from the live attenuated pathogens they contain [1].

In addition to vaccinating patients, vaccinating their environment is also recommended—a strategy known as “cocooning”. This approach aims to shield immunocompromised individuals who may be unable to receive certain vaccines, such as live vaccines, or may exhibit diminished immunogenicity from inactivated vaccines [4].

Despite the clear importance of vaccination in this high-risk population, vaccination rates among patients undergoing biologic therapy present a significant concern in clinical practice, while published data regarding this topic are limited. The primary cause behind inadequate vaccination rates appears to stem from a lack of information. This is further compounded by various factors, including skepticism regarding vaccine efficacy and potential side effects, perceptions of vaccination as unnecessary, fear of disease exacerbation, and pervasive anti-vaccination attitudes [4,5,6]. Within the medical community, healthcare providers face their own hurdles, such as the ambiguity between general practitioners and specialists regarding vaccination recommendation responsibilities, the absence of clear vaccination guidelines for patients on biologic therapies, and sporadic patient–physician interactions [4,6].

This study aims to assess vaccination coverage and identify potential barriers to vaccination among individuals on biologic agents. Moreover, it seeks to elucidate potential associations between various demographic, clinical, and behavioral factors and the adequacy of vaccination with each recommended vaccine. Additionally, this study aims to identify factors associated with the overall willingness of patients to undergo vaccination. Understanding these factors is crucial for creating better healthcare policies and interventions aiming at improving vaccination rates and mitigating the risk of vaccine-preventable diseases in this growing patient population. In this study, the vaccination status of household contacts was also assessed, to our knowledge, for the first time in such a big cohort.

Through a systematic examination of vaccination coverage, the results of this study aim to highlight the need for the development of tailored strategies that promote immunization adherence and safeguard the health and well-being of individuals receiving biologics.

## 2. Methodology

This study was conducted in accordance with the EQUATOR Network guidelines, and the manuscript was prepared following the Strengthening Reporting of Observational Studies in Epidemiology (STROBE) checklist.

### 2.1. Population

The study population included randomly enrolled adults and all pediatric patients undergoing treatment with biologics at the time, who were followed up at the two hospitals in Heraklion, Crete.

The vaccination coverage of their close contacts, which was defined as people living in the same house with them, was also studied.

### 2.2. Design and Methods

A cross-sectional study was conducted between September 2022 and February 2023. Informed consent for participation in the study was obtained from adult patients and parents of pediatric patients prior to enrolment. Inclusion criteria were the receipt of a biologic agent and willingness to participate in the study. There were no exclusion criteria apart from refusal to participate. The parents of pediatric patients were asked to present their children’s vaccination records. Adult patients were asked to recall whether they had been vaccinated with specific vaccines, and their response was cross-checked with the electronic prescription system and other available records (medical booklets and files). Vaccination against *Streptococcus pneumoniae*, influenza, Diphtheria–Tetanus–Pertussis, and COVID-19 was evaluated for adult patients, while for pediatric patients, all childhood vaccines were examined. Vaccination coverage was assessed according to National Recommendations for high-risk groups. The vaccination status of patients’ household members against influenza, Diphtheria–Tetanus–Pertussis, COVID-19, Measles–Mumps–Rubella, and Varicella was also assessed to investigate whether the recommended cocooning strategy was followed. In addition, demographics, medical history, intention to vaccinate, and reasons for undervaccination were assessed.

The study was approved by the Ethics Committee of both hospitals (University Hospital of Heraklion: Approval Code: 2530, Approval Date: 25 January 2023, Venizeleio General Hospital of Heraklion: Approval Code: 158, Approval Date: 22 November 2022) and conformed to the Declaration of Helsinki.

### 2.3. Statistical Analysis

Statistical analysis used absolute and relative frequencies to describe the study population and vaccination status for each vaccine. Comparisons between vaccinated and unvaccinated groups were performed using the χ^2^-test or Fisher’s exact test for nominal variables, the Mann–Whitney U test for ordinal variables, and the t-test or Mann–Whitney U test for quantitative variables was used, depending on data distribution. Significant parameters identified were included in a multivariate logistic regression. Similarly, this procedure was utilized to explore the association between various parameters and the willingness to be vaccinated. The results were expressed as the Adjusted Odds Ratio (AOR) and 95% Confidence Interval (CI95%) with corresponding *p*-values. The level of statistical significance for all analyses was set at α = 0.05; thus, results with a *p*-value < 0.05 (two-tailed) were considered statistically significant. Microsoft Excel 2016 and MedCalc 2006 were used for statistical analysis.

## 3. Results

### 3.1. Participants’ Characteristics

#### 3.1.1. Characteristics of Adult Patients

A total of 1143 individuals participated in the study. Among them, 446 were adult patients on biologics, 71.3% of whom were women (*n* = 318). The mean age of adult patients was 59.7 ± 13 years (median 60, range 19–89). Their 618 household contacts had a mean age of 48.9 ± 20.6 years (median 52, range 1–81). The most common diagnosis among patients was rheumatoid arthritis (46.8%, *n* = 209), and 75.6% (*n* = 337) had comorbidities. Regarding biologic treatments, 69.1% (n = 308) received intravenous agents, 27.8% (*n* = 124) received subcutaneous agents, and 3.1% (*n* = 14) received oral treatment, with Infliximab (49.7%, *n* = 153), Adalimumab (25.8%, *n* = 32), and Apremilast (28.6%, *n* = 4) being the most common in each category, respectively (Table 1).

#### 3.1.2. Characteristics of Pediatric Patients

Twenty-six (26) children and adolescents on biologics were included in the study, of which 12 (46.2%) were girls. The mean age was 11.7 (±4.1) years (median 13, range 3–18). Their household contacts consisted of 53 adults with a mean age of 44.4 (±9.3) years (median 43, range 20–75). Also, their close environment included 18 children with a mean age of 10.3 (±4.6) years (median 11.5, range 1–17). Half of the pediatric patients had Crohn’s disease (*n* = 13, 50%). Biologic agents were intravenous in *n* = 17 (65.4%) and subcutaneous in *n* = 9 (34.6%) patients, with Infliximab (*n* = 10, 38.5%) and Adalimumab (*n* = 7, 77.8%) being the main representatives. Five children (19.2%) had comorbidities, of which three had asthma and two had allergic rhinitis. Further data are shown in Table 1.

### 3.2. Vaccination Coverage of Patients

#### 3.2.1. Vaccination Coverage of Adult Patients

**Figure 1:**
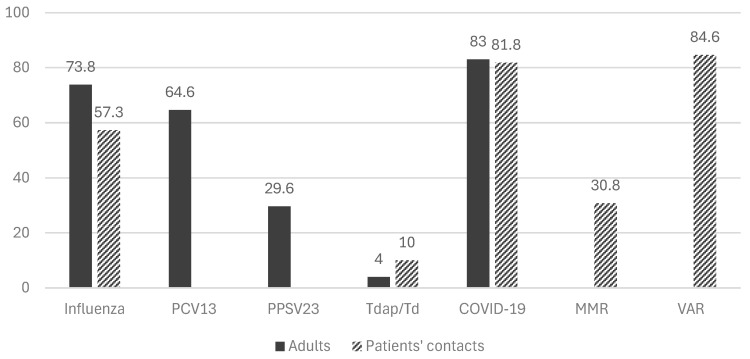
Vaccination rates of adult patients and their household contacts. **Abbreviations:** PCV13: 13-valent pneumococcal conjugate vaccine; PPSV-23: 23-valent pneumococcal polysaccharide vaccine; Tdap: adult-type Tetanus, Diphtheria, and Acellular Pertussis Vaccine; Td: Tetanus and Diphtheria Toxoid Vaccine; COVID-19: Coronavirus Disease 2019 vaccine; MMR: measles–mumps–rubella vaccine, VAR: Varicella vaccine.

Of the total of 446 adult patients enrolled in the study, 329 (73.8%) were compliant with the annual influenza vaccination. Two hundred and eighty-eight (288) patients (64.6%) were vaccinated with the pneumococcal conjugate vaccine (PCV), while 132 (29.6%) were vaccinated with at least one dose of the pneumococcal polysaccharide vaccine (PPSV) and 12 (9%) of these were vaccinated with two doses. Eighteen (4%) were vaccinated against Tetanus–Diphtheria–Pertussis (Tdap) within the last decade. Finally, 370 people (83%) were vaccinated against COVID-19, of which 29 (7.8%) had received two doses, 249 (67.3%) three doses, 68 (18.4%) four doses and only 24 (6.5%) received five doses (Figure 1).

#### 3.2.2. Vaccination Coverage of Pediatric Patients

**Figure 2:**
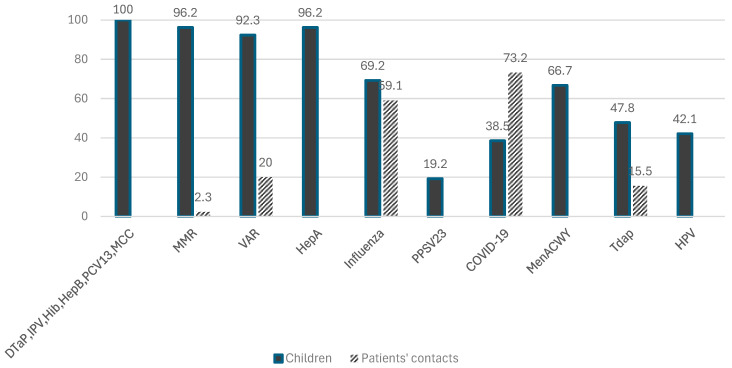
Vaccination rates of pediatric patients and their household contacts. **Abbreviations:** DTaP: Diphtheria, Tetanus, and Acellular Pertussis Vaccine; IPV: Inactivated Poliovirus Vaccine; Hib: Haemophilus influenza type b vaccine; HepB: Hepatitis B vaccine; PCV13: 13-valent pneumococcal conjugate vaccine; MCC: meningococcal group C conjugate vaccine; MMR: measles–mumps–rubella vaccine, VAR: Varicella vaccine; HepA: Hepatitis A vaccine; PPSV-23: 23-valent pneumococcal polysaccharide vaccine; COVID-19: Coronavirus Disease 2019 vaccine; MenACWY: meningococcal group A, C, W, Y conjugate vaccine; Tdap: adult-type Tetanus, Diphtheria, and Acellular Pertussis Vaccine; HPV: Human Papillomavirus Vaccine.

Regarding the standard recommended vaccines for children, all 26 patients were fully vaccinated against Diphtheria–Tetanus–Pertussis (DTaP), poliomyelitis (IPV), *Haemophilus influenza type B* (Hib), Hepatitis B (HepB), Meningococcus serogroup C (MCC) and *Streptococcus pneumoniae* with the conjugate vaccine (PCV). Five patients (19.2%) received the polysachharide vaccine against *Streptocococcus pneumonie* (PPSV), with two patients having received two doses and three patients one dose. The meningococcal ACWY (MenACWY) vaccine was age-eligible for 18 children, of whom 12 (66.7%) received it, including children on Eculizumab (*n* = 2, aged 13 and 18 years), both of whom had received two doses. The serogroup B meningococcal vaccine (MenB) had also been administered to both of these patients (two doses in one and one dose in the other). Prior to the initiation of treatment, twenty-five patients (96.2%) were vaccinated with the measles–mumps–rubella vaccine (MMR) (18 (72%) with two doses and 7 (28%) with one dose) and twenty-four (92.3%) received the Varicella vaccine (VAR), of which 19 (79.2%) patients had two doses and 5 patients (20.8%) had one dose. One child had natural immunity to chicken pox. For Hepatitis A (HepA), 25 (96.2%) children were vaccinated (22 children (88%) with two doses and 3 (12%) with one dose), and 6 (23.1%) were vaccinated against tuberculosis (BCG). Among the patients who were age-eligible to receive the following vaccines, 11 (47.8%) had received the Tdap vaccine, and 8 (42.1%) received the HPV vaccine, of whom 6 received two doses and 2 received one dose. No child was vaccinated with three doses against HPV, as recommended for patients treated with biologics. Eighteen patients (69.2%) were vaccinated with the seasonal influenza vaccine annually, and 10 (38.5%) were vaccinated with the COVID-19 vaccine (9 patients (90%) received two doses and 1 (10%) received four doses). (Figure 2)

### 3.3. Coverage Rates of Patients’ Close Contacts

#### 3.3.1. Contacts of Adult Patients

Of all the people in the household environment of adult patients (*n* = 618), 57.3% (*n* = 354) were vaccinated against influenza, 10% (*n* = 62) with Tdap, and 81.8% (*n* = 506) against COVID-19. Of the 618 people, 306 (49.5%) were born before 1970 and were, therefore, considered immune to measles. The remaining 312 (50.5%) were born after 1970 and should have received two doses of the MMR vaccine unless contraindicated. Among those born after 1970, 96 individuals (30.8%) were vaccinated against measles, while the disease history for some of the others was unclear. For Varicella, 153 individuals who were born after 1990 were considered susceptible; of these, 105 (68.6%) were vaccinated, and 29 (19%) were immune due to natural infection (Figure 1).

#### 3.3.2. Contacts of Pediatric Patients

The household members of pediatric patients included 71 people (53 adults and 18 children). Of these, 42 (59.2%) were vaccinated against influenza (35 adults, 7 children) and 52 (73.2%) were vaccinated against COVID-19 (45 adults, 7 children). For the Tdap vaccine, 8 out of 14 eligible children (57.1%) and three adults (5.7%) were vaccinated. Among the 53 adults, 10 (18.9%) were born before 1970 and were considered immune to measles, while 43 (81.1%) should have received two doses of MMR unless contraindicated. All children and one adult (2.3%) were vaccinated with MMR, and one adult had a history of natural measles. All but one child and one of five (20%) adults born after 1990 had received the Varicella vaccine (Figure 2).

#### 3.3.3. Correlation of Patients’ Characteristics with Their Vaccination Status

Univariate analyses followed by multivariate analysis incorporating the statistically significant variables from the initial results assessed the following parameters: gender, age, education level, smoking status, presence of comorbidities, route of administration of biologic agents (intravenous or subcutaneous), the number of prior biologic agents received (as an indicator of refractory disease), time since diagnosis, the duration of disease before initiating biologic therapy, and the time since the initiation of treatment. Similarly, for pediatric patients, parameters including age, comorbidities, the route of biologic agent administration, time since diagnosis, the duration of disease before biologic agent initiation, and time since initiating biologic therapy were explored concerning vaccination beyond the standard childhood immunization program, e.g., influenza, PPS, and COVID-19 vaccines. The results of adult patients are shown below for each vaccine.

Influenza: Women had a significantly higher vaccination rate compared to men (77.3% vs. 64.8%, *p* = 0.007), and vaccinated individuals were older on average (61.14 ± 13.08 years vs. 55.69 ± 11.95 years, *p* < 0.001). Multivariate logistic regression analysis identified female gender (OR = 1.84, CI95%: 1.16–2.9, *p* = 0.007) and age (OR = 1.03 per 1-year, CI95%: 1.02–1.05, *p* <0.001) as significant independent predictors of influenza vaccination.

PCV13: The vaccination rate was higher in women (69.1% vs. 53.1% in men, *p* = 0.001), of older ages (62.5 ± 11.36 vs. 54.5 ± 14.21 years, *p* < 0.001), and in people with comorbidities (69.4% vs. 49.5%, *p* < 0.001). Lower education levels were associated with better vaccination coverage (elementary school graduates, 68.9%; middle school graduates, 69.3%; high school graduates, 58.4%; and tertiary education graduates, 57.9% with *p* = 0.037). Logistic regression identified gender (OR = 2.18, CI95%: 1.37–3.46, *p* = 0.001) and age (OR = 1.05, CI95%: 1.03–1.08, *p* <0.001) as significant independent predictors of PCV13 vaccination.

PPSV: Significantly higher vaccination rates were seen in women (35.5% vs. 14.8%, *p* < 0.001) and the average age of vaccinated individuals (62.8 ± 12.33 years) was older than that of non-vaccinated individuals (58.4 ± 13.07 years, *p* < 0.001). The vaccination rate was also statistically significantly higher in non-smokers (34.6% vs. 18.6%, *p* = 0.001) and in patients receiving intravenous biologics (32.8%) compared to those receiving subcutaneous biologics (21.8%, *p* = 0.023). Logistic regression confirmed gender (OR = 3.26, CI95%: 1.83–5.81, *p* < 0.001), age (OR = 1.02, CI95%: 1.00–1.04, *p* = 0.018), and receiving intravenous agents (OR = 1.71, CI95%: 1.03–2.83, *p* = 0.038) as significant independent predictors of PPSV vaccination.

Tdap: Higher rates of vaccination, albeit extremely low, were observed in the male sex (8.6% vs. 2.2%, *p* = 0.002), in younger ages (the average age of vaccinated individuals was 49.44 ± 15.89 years, vs. 60.14 ± 12.71 years in non-vaccinated individuals, *p* = 0.001) in people with higher education (vaccinated graduates of elementary school and middle school 0%, high school 5.1%, tertiary 17.4%, *p* < 0.001), in patients who had a shorter history of receiving biologic agents (*p* = 0.024), and in the individuals whose diagnosis was more recent (the mean time since diagnosis in the vaccinated group was 10.89 ± 14.64 years vs. 13.73 ± 9.96 years in the unvaccinated group, *p* = 0.017). Multivariate logistic regression analysis identified the gender (OR = 0.09, CI95%: 0.03–0.29, *p* < 0.001), education (OR = 0.16, CI95%: 0.05–0.55, *p* = 0.004), and history of prior recipients of biologic agents (OR = 0.55, CI95%: 0.31–0.98, *p* = 0.043) as significant independent predictors of Tdap vaccination.

COVID-19: The vaccination rate was higher in females than in males (86.5% vs. 74.2%, *p* = 0.002), and the mean age of the vaccinated individuals was older (60.88 ± 13.26 vs. 53.99 ± 9.91 years, *p* < 0.001). Also, the vaccination rate was higher in patients without comorbidities (86.1% vs. 73.4%, *p* = 0.002). Multivariate analysis confirmed gender (OR = 2.19, CI95%: 1.30–3.69, *p* = 0.003) and age (OR = 1.04, CI95%: 1.01–1, 06, *p* = 0.001) to be significant independent predictors of COVID-19 vaccination.

In pediatric patients, no significant associations with vaccination coverage were found.

#### 3.3.4. Factors Associated with Willingness to Be Vaccinated

Factors influencing willingness to be vaccinated varied across adult and pediatric populations. Among adults, 77.3% were willing to receive missed vaccines, while 22.7% were not. Univariate analyses showed that women were significantly more willing than men (82.7% vs. 64%, *p* < 0.001), while the average age of patients who expressed willingness to be vaccinated was statistically significantly higher (61.08 ± 13.04 vs. 55.02 ± 11.78 years, *p* < 0.001). Patients with comorbidities appeared more likely to accept vaccination (79.7% vs. 69.7%, *p* = 0.029). Logistic regression confirmed gender (OR = 2.7, CI95%: 1.68–4.33, *p* < 0.001) and age (OR = 1.04, CI95%: 1.02–1.06, *p* < 0.001) as statistically significant predictors of willingness to be vaccinated. Conversely, among pediatric patients, 80.8% of parents expressed willingness to vaccinate their children with missed vaccines, but no statistically significant correlations were found between parental willingness and the examined parameters.

### 3.4. Reasons of Undervaccination

#### 3.4.1. Reasons of Undervaccination of Adult Patients

Out of the 446 adults included in the study, only 6 (1.3%) were fully vaccinated with all recommended vaccines. Among the rest, 69.5% (*n* = 306) cited a lack of knowledge, as they had not received a recommendation from their physician. Additionally, 9.8% (*n* = 43) feared side effects, 4.5% (*n* = 20) were concerned about potential disease outcomes, 7.7% (*n* = 34) had a negative attitude towards vaccination without specifying a reason, and 7% (*n* = 31) considered vaccination unnecessary. Lastly, 1.4% (*n* = 6) had received a recommendation from their doctor but neglected to receive the vaccine.

#### 3.4.2. Reasons of Undervaccination of Pediatric Patients

Only one child was fully vaccinated with all age-appropriate vaccines. Among the parents of the other children, 60% (*n* = 15) reported not receiving a recommendation from their pediatrician, 20% (*n* = 5) feared side effects, and 16% (*n* = 4) considered the missed vaccines unnecessary. Two parents (8%) delayed or neglected vaccinations due to the COVID-19 pandemic, while one (4%) feared disease exacerbation after vaccination. Lastly, the parents of 1 child (4%) on hemodialysis cited scheduling difficulties due to the timing of hemodialysis and frequent viral infections.

## 4. Discussion

The present study assessed vaccination coverage and barriers to vaccination among individuals on biologic agents, examining how factors like age, gender, education, smoking, and comorbidities affect vaccination adequacy and willingness. This study also evaluated the vaccination status of household contacts.

According to the results, the majority of adult patients were vaccinated against COVID-19 and influenza. Fewer than two-thirds of patients had received the pneumococcal conjugate vaccine, while a much smaller proportion had received the pneumococcal polysaccharide vaccine and the adult-type Diphtheria–Tetanus–Pertussis vaccine within the last decade.

The vaccination rate in the household environment of adult patients was low for all vaccines examined, except for vaccination against COVID-19.

The moderate vaccination rate for Pneumococcus demonstrated in the present study is consistent with other studies conducted in immunocompromised patients, including patients on biologic therapy. In a cohort of 499 patients on biologics, the vaccination coverage for Pneumococcus was 49% [7]. The same vaccination rate (49%) was found in two other studies, one including a cohort of 208 patients under biologic therapy [8] and a large cohort of patients with autoimmune diseases who were treated with immunosuppressive drugs, including 1378 patients on biologic agent [9]. In fact, being treated with a biologic agent was negatively associated with pneumococcal vaccination [9,10,11]. Additionally, when patients with dermatological diseases receiving biologic therapy were evaluated, only 17% received the pneumococcal vaccine [12]. In contrast, Fernández-Prada et al. showed high coverage with PCV13 (80.2%), PPSV23 (77.9%), or both PCV13 + PPSV23 (75.2%) among rheumatology patients receiving biologic therapy [13].

Regarding influenza vaccination, the results of our study show increased vaccination coverage compared to a study of 208 patients on biologics (28% vaccinated) [8] but are similar to another study of patients on biologic therapy, in which 78.8% of patients were vaccinated against the flu [13]. A recent publication from Greece showed an increase in the vaccination coverage of patients with rheumatic diseases during the pandemic from 76% to 83% [14], while another Greek study conducted 2 years before the aforementioned study showed a vaccination rate of 54% for influenza in patients with rheumatoid arthritis [15].

The high vaccination rate against COVID-19 found in the present study is consistent with other studies, in which vaccination rates reach up to 94.4% [16,17,18,19] and can be attributed to the fact that this vaccine was compulsory.

Regarding vaccination coverage in the environment of patients, the present study is the first to address this issue to our knowledge. Our findings show that people in the environment of immunosuppressed patients on biologics are suboptimally vaccinated. If we take into account that many of the people in the patients’ environment may have a variety of other indications for vaccination, i.e., age, comorbidities, etc., we conclude that the percentage of those who are vaccinated due to adherence to the cocooning strategy is extremely small.

Pediatric patients on biologics were sufficiently vaccinated with the vaccines of the basic immunization schedule but inadequately covered with adolescence and group-specific vaccines. A cross-sectional study from a Russian tertiary center, including 82 juvenile idiopathic arthritis patients on biologics found a vaccination rate of 61% for MMR and 31% for Diphtheria [11]. A scoping review of various studies conducted in children with autoimmune and autoinflammatory diseases treated with immunosuppressive regimes, including biologics, showed varying vaccination coverage rates by vaccine, but overall they were lower than those found in the present study (DTaP-IPV-Hib-HepB 67–99.3%, pneumococcal 7–42%, MMR 86–92%, VAR 18.4–74%, MCC 9.4–76%, HPV 5.9%, influenza 1.7–84.3%) [3].

The differences observed in different studies reflect the design of the studies, the vaccination program, and health systems of different countries, and cultural differences, as well as increasing awareness about vaccinations nowadays.

The vaccination coverage rates of family members of pediatric patients were moderate for influenza (59.2%) and COVID-19 (73.2%) and very low for MMR-VAR (26.2%) and Tdap (16.4%). This suggests that the recommendation for vaccination in the setting of immunocompromised children is likely neglected as a strategy to protect them.

Another interesting finding of the present study is that there were several deviations from the recommended vaccination schedule. These include delays in initiating vaccination, sometimes occurring years after starting biologic therapy, the administration of more than one dose of PCV13, incorrect sequencing of PCV13 and PPSV23 administration, administering vaccines at intervals much longer than recommended, irregular rather than annual influenza vaccinations and receiving fewer doses than recommended.

Of all the vaccines, except for the vaccine against COVID-19, the flu vaccine seems to be the most well-known one and the one with the widest public acceptance. In contrast, pneumococcal and tetanus vaccines are not widely known and are used by patients only after recommendation from their doctor. This is reflected in our study, where the majority of patients who were not vaccinated with these vaccines stated a lack of knowledge since they had not received a recommendation from their doctor for vaccination. This has been shown by published studies that have highlighted the lack of recommendation by the attending physician as the primary cause of insufficient vaccination coverage [8,14,20]. The role of healthcare professionals is crucial in promoting vaccinations for both the general population and vulnerable patient groups. Educating healthcare professionals in different specialties is of the utmost importance for the efficient implementation of special vaccine recommendations for high-risk groups of patients. Other causes that have emerged in the literature and which were identified in the present study are the view that vaccination is unnecessary, the fear of possible negative effects of vaccination, and the prevailing fear of disease exacerbation, denial, and neglect [4,14,20,21,22]. All patients should have the opportunity to discuss their fears and misconceptions about vaccines with their physicians, and any potential side effects should be addressed prior to immunization in accordance with good clinical practices. Further reasons reported in the literature were patients’ doubts about the efficacy of immunization and, interestingly, discouragement from health workers [11,12,23].

Also consistent with findings from prior research, this study observed higher rates of influenza and pneumococcal vaccination among the elderly [10,14,24,25]. It is plausible that the elevated coverage in this age group is driven, on the one hand, by age itself serving as an indication for these vaccines, and on the other hand, by the more frequent contact with medical services rather than solely by the immunosuppression status. Additional insights from the literature, including the inverse relationship between vaccination coverage and the receipt of biologic agents, as well as the positive correlation between comorbidities and higher coverage, suggest that the biologic agent treatment may constitute an overlooked risk factor for vaccine-preventable infections [9]. Moreover, the finding that female sex, advanced age, and the presence of comorbidities were positively associated with willingness to be vaccinated is consistent with previous research [18]. These findings underscore the fact that the incentive for vaccination in this group may not solely stem from the risk of serious infections due to immunosuppression and suggest that vaccination recommendations might neglect younger and otherwise healthy individuals.

A major advantage of the study is the large sample size of adult patients and the large total number of subjects included. Furthermore, to our knowledge, it is the first study to assess the cocooning of patients on biologic therapy. In addition, the study included patients on biologic treatment irrespective of disease to examine the receipt of a biologic agent as an independent factor that constitutes an indication for vaccination.

A limitation of this study is the potential inaccuracy of information provided by patients and parents, primarily due to recall biases. This includes possible errors in recalling vaccination statuses and details of their conversations with physicians, particularly regarding vaccine-related advice. However, the first was largely overcome through the verification of information from medical files and the electronic prescription system. Also, the patients of the present study were followed at tertiary centers, and their care and behaviors may be different from patients treated in the private sector. Lastly, the sample number of pediatric patients is too small to draw firm conclusions, yet it represents the total number of children who were treated with biologic therapy in the study area at that time.

## 5. Conclusions

The results of the present study show that vaccination coverage rates of patients undergoing treatment with biologic agents and their environment is suboptimal and is a key preventive measure that needs to be strengthened. For this purpose, it is considered necessary to take appropriate actions that focus on improving information about the indications and benefits of vaccination and promoting knowledge, primarily among doctors but also among patients and their close contacts.

## Figures and Tables

**Table 1:** Medical conditions and biologic agents of the study participants, both adults and children.

Category	Condition/Treatment	Adults *n* (%)	Children *n* (%)
**Total Patients**		**446 (100%)**	**26 (100%)**
**Medical Condition**	Rheumatoid Arthritis (RA)	209 (46.8)	-
	Spondyloarthritis (SpA)	65 (14.5)	-
	Ankylosing Spondylitis (AS)	55 (12.3)	-
	Systemic Lupus Erythematosus (SLE)	47 (10.5)	-
	Psoriatic Arthritis (PsA)	48 (10.8)	1 (3.8)
	Enteropathic Arthritis	16 (3.6)	-
	Juvenile Idiopathic Arthritis (JIA)	7 (1.5)	3 (11.5)
	Scleroderma	5 (1.1)	1 (3.8)
	Temporal Arteritis	2 (0.4)	-
	SAPHO Syndrome	1 (0.2)	-
	Adamantiades-Behçet disease	1 (0.2)	-
	Undifferentiated Connective Tissue Disease (UCTD)	1 (0.2)	-
	Still’s Disease	1 (0.2)	-
	Crohn’s Disease	-	13 (50)
	Inflammatory Bowel Disease (IBD)	-	2 (7.7)
	Systemic JIA (SJIA)	-	1 (3.8)
	Atypical Hemolytic Uremic Syndrome (aHUS)	-	2 (7.7)
	Nephrotic Syndrome	-	1 (3.8)
	Focal Segmental Glomerulosclerosis (FSGS)	-	1 (3.8)
	Chronic Recurrent Multifocal Osteomyelitis (CRMO)	-	1 (3.8)
**Intravenous Biologic Agent**		308 (69.1)	17 (65.4)
	Infliximab	153 (49.7)	10 (38.5)
	Abatacept	73 (23.7)	-
	Tocilizumab	48 (15.6)	1 (3.8)
	Belimumab	30 (9.7)	-
	Anifrolumab	3 (1.0)	-
	Rituximab	1 (0.3)	2 (7.7)
	Vedolizumab	-	2 (7.7)
	Eculizumab	-	2 (7.7)
**Subcutaneous Biologic Agent**		124 (27.8)	9 (34.6)
	Adalimumab	32 (25.8)	7 (77.8)
	Etanercept	25 (20.2)	1 (11.1)
	Secukinumab	21 (16.9)	-
	Certolizumab	13 (10.5)	-
	Golimumab	12 (9.7)	-
	Tocilizumab	9 (7.3)	-
	Abatacept	6 (4.8)	-
	Anakinra	4 (3.2)	1 (11.1)
	Infliximab	1 (0.8)	-
	Belimumab	1 (0.8)	-
**Oral Biologic Agent**		14 (3.1)	-
	Tofacitinib	8 (57.1)	-
	Apremilast	4 (28.6)	-
	Upadacitinib	1 (7.1)	-
	Baricitinib	1 (7.1)	-

## Data Availability

The original contributions presented in this study are included in the article. Further inquiries can be directed to the corresponding author(s).
